# Selective regulation of MAP kinases and Chemokine expression after ligation of ICAM-1 on human airway epithelial cells

**DOI:** 10.1186/1465-9921-7-12

**Published:** 2006-01-23

**Authors:** Thomas M Krunkosky, Carla L Jarrett

**Affiliations:** 1 Department of Anatomy & Radiology, College of Veterinary Medicine, University of Georgia, Athens, GA 30602, USA

## Abstract

**Background:**

Intercellular adhesion molecule 1 (ICAM-1) is an immunoglobulin-like cell adhesion molecule expressed on the surface of multiple cell types, including airway epithelial cells. It has been documented that cross-linking ICAM-1 on the surface of leukocytes results in changes in cellular function through outside-inside signaling; however, the effect of cross-linking ICAM-1 on the surface of airway epithelial cells is currently unknown. The objective of this study was to investigate whether or not cross-linking ICAM-1 on the surface of airway epithelial cells phosphorylated MAP kinases or stimulated chemokine expression and secretion.

**Methods:**

The human lung adenocarcinoma (A549) cells and primary cultures of normal human bronchial epithelial (NHBE) cells were used in these studies. To increase ICAM-1 surface expression, cultures were stimulated with TNFα to enhance ICAM-1 surface expression. Following ICAM-1 upregulation, ICAM-1 was ligated with a murine anti-human ICAM-1 antibody and subsequently cross-linked with a secondary antibody (anti-mouse IgG(ab')_2_) in the presence or absence of the MAP kinase inhibitors. Following treatments, cultures were assessed for MAPK activation and chemokine gene expression and secretion. Control cultures were treated with murine IgG1 antibody or murine IgG1 antibody and anti-mouse IgG(ab')_2 _to illustrate specificity. Data were analyzed for significance using a one-way analysis of variance (ANOVA) with Bonferroni post-test correction for multiple comparisons, and relative gene expression was analyzed using the 2^-ΔΔC^T method.

**Results:**

ICAM-1 cross-linking selectively phosphorylated both ERK and JNK MAP kinases as detected by western blot analysis. In addition, cross-linking resulted in differential regulation of chemokine expression. Specifically, IL-8 mRNA and protein secretion was not altered by ICAM-1 cross-linking, in contrast, RANTES mRNA and protein secretion was induced in both epithelial cultures. These events were specifically inhibited by the ERK inhibitor PD98059. Data indicates that ICAM-1 cross-linking stimulates a synergistic increase in TNFα-mediated RANTES production involving activation of ERK in airway epithelial cells.

**Conclusion:**

Results demonstrate that cytokine induced ICAM-1 on the surface of airway epithelial cells induce outside-inside signaling through cross-linking ICAM-1, selectively altering intracellular pathways and cytokine production. These results suggest that ICAM-1 cross-linking can contribute to inflammation in the lung via production of the chemokine RANTES.

## Background

The airway epithelium lining the airways functions as a protective barrier from inhaled particulates and aerosols from the external environment. Additionally, it regulates leukocyte trafficking into the airway lumen through adhesion molecules and cytokine responses. Therefore, in the inflamed airway, epithelial cells can function as both ''target'' and ''effector'' cells. As target cells, they are influenced by exogenous inflammatory agents. As effector cells, they produce and release inflammatory mediators. Many of these cellular events are regulated by interactions through adhesion molecules as well as soluble factors such as chemokines.

Intercellular adhesion molecule-1 (ICAM-1) is a 95 kDa surface glycoprotein [belonging to the immunoglobulin supergene family] that has been detected on a variety of cell types, including human airway epithelium [1]. ICAM-1 is involved in cell-to-cell interactions and microbial pathogenesis. ICAM-1 has also been suggested to participate in cell signaling through outside-inside signaling events in several different cell types [2]. Interestingly, it has not been determined whether ICAM-1 functions as a signaling molecule to transmit biochemical signals in airway epithelial cells.

Previously [3], we have demonstrated that the cytokine TNFα, upregulates both gene and surface expression of ICAM-1 in airway epithelial cells in vitro. It has been well documented that airway epithelial cells produce chemokines, which are involved in airway inflammation [4-6]. Both RANTES (regulated on activation normal T cell expressed and secreted) and interleukin-8 (IL-8) are chemokines that are secreted by the epithelium and play an important role in asthmatic airways through the recruitment of inflammatory cells by functioning as chemo-attractant [7-11]. High levels of RANTES and interleukin-8 in nasal and bronchial mucosa contribute to the massive recruitment of leukocytes enhancing inflammation in the airway.

One of the signaling pathways implicated in regulating both IL-8 and RANTES expression is the mitogen-activated protein (MAP) kinase cascade [12,13]. Many extracellular stimuli have been shown to elicit specific biologic responses through activation of MAP kinases [14]. The MAP kinase superfamily has been molecularly characterized into three groups which include: extracellular signal-regulated kinase p42 p44 (ERK), p38 MAP kinase (HOG), and JNK/SAPK (c-jun N-terminal kinase/stress activated protein kinase). The MAP kinase cascade has been demonstrated to play a central role in airway remodeling [15,16] and plays an important role in the induction of several chemokines through various environmental factors [17,18].

This study examined the effects of cross-linking ICAM-1 on outside-inside signaling in human airway epithelial cells. Specifically, we investigated whether ligation of ICAM-1 on airway epithelial cells resulted in activation of ERK, p38 and JNK. Additionally, we examined whether cross-linking ICAM-1 induced IL-8 and RANTES gene expression and protein secretion through activation of MAP kinases. Utilizing both a human lung adenocarcinoma cell line (A549) and primary cultures of normal human bronchial epithelial (NHBE) cells, data representing the normal airway epithelium did not respond to ICAM-1 cross-linking. In contrast, cultures that were stimulated by the cytokine TNFα to increase ICAM-1 surface expression, resulted in selective activation of the MAP kinases ERK and JNK. In addition, cross-linking ICAM-1 on cells pre-exposed to TNFα differentially stimulated IL-8 and RANTES gene expression and secretion. Our results suggest that cytokine induced ICAM-1 on the surface of airway epithelial cells selectively activate both MAP kinases and chemokines through ICAM-1 cross-linking. Additionally, cross-linking ICAM-1 induced RANTES production appears mediated through an ERK-dependent pathway.

## Materials and methods

### Sources of reagents

All the reagents were purchased from Sigma Chemical Co. (St. Louis, MO) unless otherwise stated. Human recombinant TNFα (hrTNFα; 0.015–150 ng/ml [Spec. Activity = 2.86 × 10^7 ^U/mg]) and RANTES sandwich ELISA kit were purchased from R&D Systems (Mineapolis, MN). Ham's F-12K medium, fetal bovine serum (FBS), penicillin and streptomycin were purchased from GIBCO BRL (Rockville, MD). Monoclonal antibody mouse anti-human CD54 (Clone 6.5B5) was purchased from DAKO Corporation (Carpinteria, CA). Negative control monoclonal antibody (IgG1) was purchased from Becton Dickinson (San Jose, CA). Polyclonal antibody goat anti-mouse IgG Fc, γ specific was purchased from ICN Biomedical (Costa Mesa, CA). The selective MEK inhibitor PD98059 and the JNK inhibitor (JNK inhibitor-1) was purchased from Calbiochem (La Jolla, CA).

### Culture of cells

#### NHBE culture

Expansion, cryopreservation and culturing of NHBE cells in an air-liquid interface system were performed as previously described [3]. Briefly, NHBE cells (Clonetics, San Diego, CA) were seeded into vented T75 tissue culture flasks (500 cells/cm^2^) until cells reached 75–80% confluence. Cultures were dissociated with trypsin/EDTA and cultured in an air-liquid interface system initiated by seeding NHBE cells (passage-2, 2 × 10^4 ^cells/cm^2^) onto Transwell-clear culture inserts (24.5 mm, 0.45 mm pore size; Costar, Cambridge, MA) that were thin-coated with rat tail collagen, type I (Collaborative Res., Bedford, MA). Cells were cultured submerged for the first 5–7 days. At that time, the air-liquid interface was created by removing the apical medium and feeding cells with medium on their basal surface only. The apical surface of the cells was exposed to a humidified 95% air/5% CO_2 _environment. Medium beneath the cells was changed daily thereafter [3]. Cells were cultured for an additional 14 days in air-liquid interface, for a total of 21 days in culture.

#### A549 culture

The A549 tumor-cell line, initiated from a human alveolar cell carcinoma (ATCC CCL-185, Rockville, MD) was cultured in Ham's F-12K medium supplemented with 10% FBS, penicillin (100 U/ml), and streptomycin (100 μg/ml). Cells were grown at 37°C in a humidified atmosphere containing 5% CO2 on 6-well plastic culture plates. Cultures were utilized upon reaching confluency.

#### Measurement of chemokine secretion

Concentrations of IL-8 and RANTES in the culture supernatants were quantified by using a commercially available sandwich ELISA kits (R&D Systems, Minneapolis, MN). All samples were assayed in triplicate.

#### Western blot analysis of MAP kinases

Analysis of threonine and tyrosine phosphorylation of MAP kinases were performed using an anti-phosphorylated threonine and tyrosine p42/p44, JNK and p38 MAP kinase antibodies (New England Bio Labs, Beverly, MA), specific for active p42/p44, JNK and p38 MAP kinase. Following treatments, cells were lysed in lysis buffer (50 mM HEPES, 150 mM NaCl, 1% Triton X-100, 1 mM AEBSF, 20 μg/ml aprotinin, and 20 μg/ml leupeptin) and clarified by centrifugation at 13,000 rpm for 10 minutes. Harvested protein concentrations were determined using the Bradford dye-binding procedure (Bio-Rad Protein Assay; Bio-Rad, Richmond, CA). Following quantification, samples containing 25 μg of protein were separated by 12% SDS-polyacrylamide gel electrophoresis, electrophoretically transferred to a membrane, and incubated with specific antibodies. Following antibody treatments, blots were incubated with horseradish peroxidase (HRP)-conjugated secondary antibody (Pierce Biotechnology, Rockford, IL). Blots were then treated with SuperSignal West Dura extended duration chemiluminescence substrate solution (Pierce Biotechnology, Rockford, IL) and analyzed using a Fluor-S Max2 (Bio-Rad, Richmond, CA) MultiImager system and associated software. Blots were stripped and reprobed using phosphorylation-state independent antibodies for p42/p44, JNK and p38 MAP kinase (New England Bio Labs, Beverly, MA) to determine total MAP kinase levels.

#### Measurement of chemokine mRNA by real time PCR

Total mRNA was harvested with the Absolutely RNA™ RT-PCR Miniprep kit (Stratagene, La Jolla, CA) according to the manufacturer's protocol. Chemokine gene expression was quantified in a two-step reverse transcription-polymerase chain reaction (RT-PCR). Complimentary DNA was reverse transcribed from total RNA samples (0.625 μg/50 μl) using random hexamers from the TaqMan RT reagents (Applied Biosystems, Foster City, CA). PCR products were synthesized from cDNA (22.5 ng/20 μl) using the TaqMan universal PCR master mix and Assays on Demand ™ gene expression reagents for human RANTES and IL-8 (Assay ID:Hs00174575_m1 and Assay ID: Hs00174103_m1, Applied Biosystems, Foster City, CA). Measurements were done using the ABI Prism 7900HT sequence detection system according to the manufacturer's protocol. As an endogenous control for these PCR quantification studies, 18S ribosomal RNA gene expression was measured using the TaqMan ribosomal RNA control reagents (Applied Biosystems, Foster City, CA). Results represent normalized IL-8 and RANTES mRNA amounts relative to control cultures using the 2^-ΔΔC^T method [19]. Each experiment was repeated in triplicate.

#### Stimulation and cross-linking of ICAM-1 on airway epithelial cultures

We have previously published that stimulation of airway epithelial cells in vitro with TNF α enhances ICAM-1 surface expression [3,20]. Therefore, to increase ICAM-1 surface expression, all experimental cultures were stimulated with 50 ng/ml TNF α for two hours followed by an incubation in medium alone for 18 hours to increase ICAM-1 expression prior to cross-linking. To cross-link ICAM-1 on the surface of both A549 and NHBE cells, cultures were incubated with 15 ug/ml anti-ICAM-1 mAb at 37°C for 30 minutes. Following incubation, cultures were washed two times with 0.1% FBS medium and subsequently incubated with 50 ug/ml anti-mouse IgG F(ab')_2_. To demonstrate specificity for ICAM-1 cross-linking, cultures were exposed to the following conditions. Cultures were incubated with 0.1% FBS medium alone, anti-mouse IgG F(ab')2 alone, control anti-mouse IgG1 alone, control anti-mouse IgG1 subsequently incubated with 50 ug/ml anti-mouse IgG F(ab')2, or anti-VCAM-1 mAb subsequently incubated with 50 ug/ml anti-mouse IgG F(ab')2 to demonstrate that the response was specifically due to crosslinking the ICAM-1 molecule on the surface of the cells. None of the combinations of antibodies altered either IL-8 or RANTES mRNA or protein secretion. This method of cross-linking ICAM-1 on the surface of cells has been widely used in multiple cell types [21-24].

#### Effect of cross-linking ICAM-1 on MAP kinase phosphorylation and chemokine gene expression and secretion

To investigate a potential role for cross-linking ICAM-1 in stimulating phosphorylation of MAP kinases, ICAM-1 was cross-linked for 5, 10, 15, and 30 minutes. Cells were then rinsed twice in PBS, and total protein was harvested and assayed for phosphorylation of MAP kinases by western blot.

To investigate the role for cross-linking ICAM-1 in chemokine steady-state mRNA expression, ICAM-1 was cross-linked for 1, 2, and 4 hours. Following cross-linking, cells were rinsed in PBS and total RNA was harvested and assayed by RT-PCR analysis. To investigate whether the effect of cross-linking ICAM-1 stimulated chemokine secretion, cells were cross-linked for ICAM-1 for 1, 2, 4, 6, and 12 hours. Following cross-linking, cell supernatants were collected and assayed for both RANTES and IL-8 by ELISA.

To address the effect of the MAP kinase inhibitors on ICAM-1 induced events, cells were pre-incubated for 30 minutes in the presence or absence of MAP kinase inhibitors followed by cross-linking of ICAM-1 for 10 minutes for ERK phosphorylation and 30 minutes for JNK activation. Cells were then rinsed twice in PBS, and samples were harvested and assayed for phosphorylation of MAP kinases by western blot. For chemokine gene and surface expression, cells were pre-incubated for 30 minutes with MAP kinase inhibitors followed by cross-linking ICAM-1 for 2 hours for chemokine steady state mRNA and 6 hours for chemokine secretion. RNA and cell supernatants were subsequently harvested for RT-PCR and ELISA.

### Statistical analysis

Data were analyzed for significance using a one-way analysis of variance (ANOVA) with Bonferroni post-test correction for multiple comparisons [25]. Data were considered significant at p < 0.05. Analysis of relative gene expression from real time PCR data were analyzed using the 2^-ΔΔC^T method [19].

## Results

### Cross-linking ICAM-1 selectively stimulates phosphorylation of p42, p44 and JNK MAP kinase in airway epithelial cells in vitro

**Figure 1:**
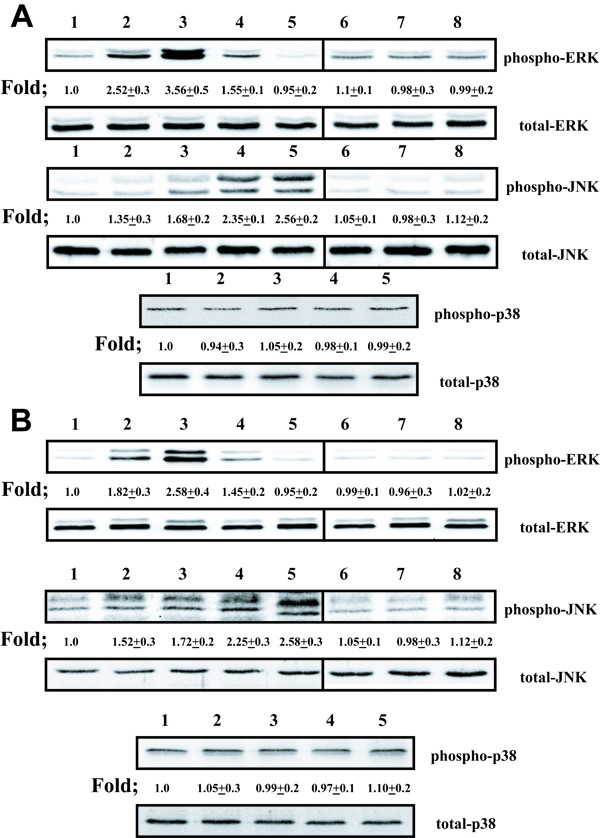
**Cross-linking of ICAM-1 induces phosphorylation of MAP kinases in airway epithelial cells. (Figure A. A549 cells) (Figure B. NHBE cells)**. **Lane 1 **represents cultures that were cross-linked with control IgG1 mAb and anti-mouse IgG F(ab')_2 _for 10 min. Lanes 2–5 represent cultures that were cross-linked with anti-ICAM-1 mAb and anti-mouse IgG F(ab')_2 _for 5 min (**lane 2**), 10 min (**lane 3**), 15 min (**lane 4**), and 30 min (**lane 5**). **Lane 6 **represents cultures that were exposed with anti-ICAM-1 mAb alone for 10 min. **Lane 7 **represents cultures that were exposed with anti-mouse IgG F(ab')_2 _mAb alone for 10 min. **Lane 8 **represents cultures that were exposed to anti-VCAM-1 mAb and anti-mouse IgG F(ab')_2 _for 10 min. Fold increase in amounts of phosphorylated MAP kinase proteins as indicated above are expressed as the mean ± SD in three different experiments.

Utilizing western blot analysis, we investigated the effect of ICAM-1 cross-linking on the phosphorylation of p42/ p44, JNK, and p38 MAP kinase. ICAM-1 cross-linking selectively stimulated phosphorylation of p42/p44 and JNK but not p38 in both A549 cells (Fig. 1a) and NHBE cells (Fig 1b) in vitro. The phosphorylation of p42/ p44 MAP kinase was maximal within 10 minutes after stimulation and gradually returned to baseline 30 minutes after treatment. The phosphorylation for JNK MAP kinase was maximal within 30 minutes of treatment. The control antibodies: (anti-mouse IgG1 and subsequently anti-mouse IgG F(ab')_2_) and (anti-VCAM-1 mAb subsequently incubated with 50 ug/ml anti-mouse IgG F(ab')_2_), did not phosphorylate p42/ p44, JNK, or p38 MAP kinase in either A549 cells or NHBE cells. Interestingly, cells that were not pre-stimulated with TNFα to increase ICAM-1 surface expression [3], did not phosphorylate p42/ p44 and JNK when treated with antibodies to cross-link ICAM-1 (data not shown).

### Cross-linking ICAM-1 selectively increases chemokine mRNA expression in airway epithelial cells in vitro

**Figure 2:**
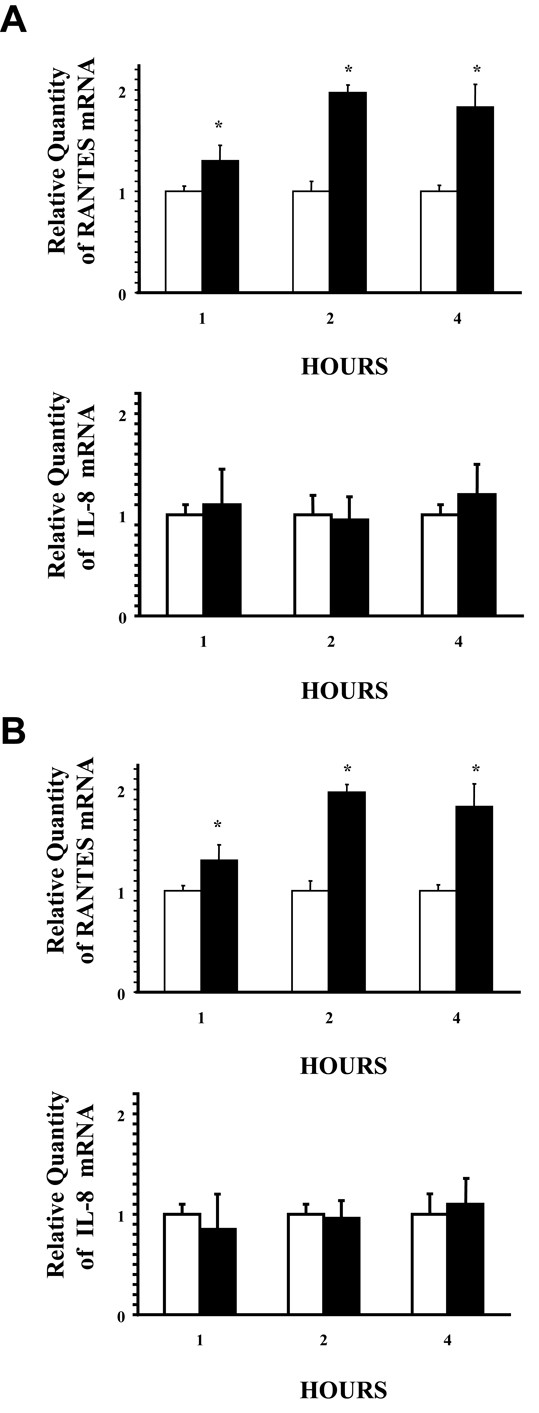
**Selective induction of chemokine mRNA by airway epithelial cultures cross-linked with ICAM-1. (Figure A. A549 cells) (Figure B. NHBE cells)**. Cultures were incubated with control IgG1 mAb and anti-mouse IgG F(ab')_2 _(**white columns**) or anti-ICAM-1 mAb and anti-mouse IgG F(ab')_2 _(**black columns**) for the indicated times before RNA was isolated for real time RT-PCR analysis of both RANTES and IL-8 message (40 PCR cycles). Messenger RNA of the 18s ribosomal gene was amplified under the same conditions and was used as the internal control. (n = 6; * = significantly different from control; p < 0.05).

To investigate the effect of ICAM-1 cross-linking on chemokine gene expression, we employed real time RT-PCR. ICAM-1 cross-linking for 1, 2 and 4 hours stimulated RANTES gene expression, however, had no effect on IL-8 gene expression in both A549 cells (Fig 2a) and NHBE cells (Fig 2b) in vitro. Cultures that were not pretreated with TNFα to increase ICAM-1 surface expression did not stimulate either chemokine gene expression when treated with antibodies to cross-link ICAM-1 (data not shown).

### Cross-linking ICAM-1 selectively stimulates chemokine secretion in airway epithelial cells in vitro

**Figure 3:**
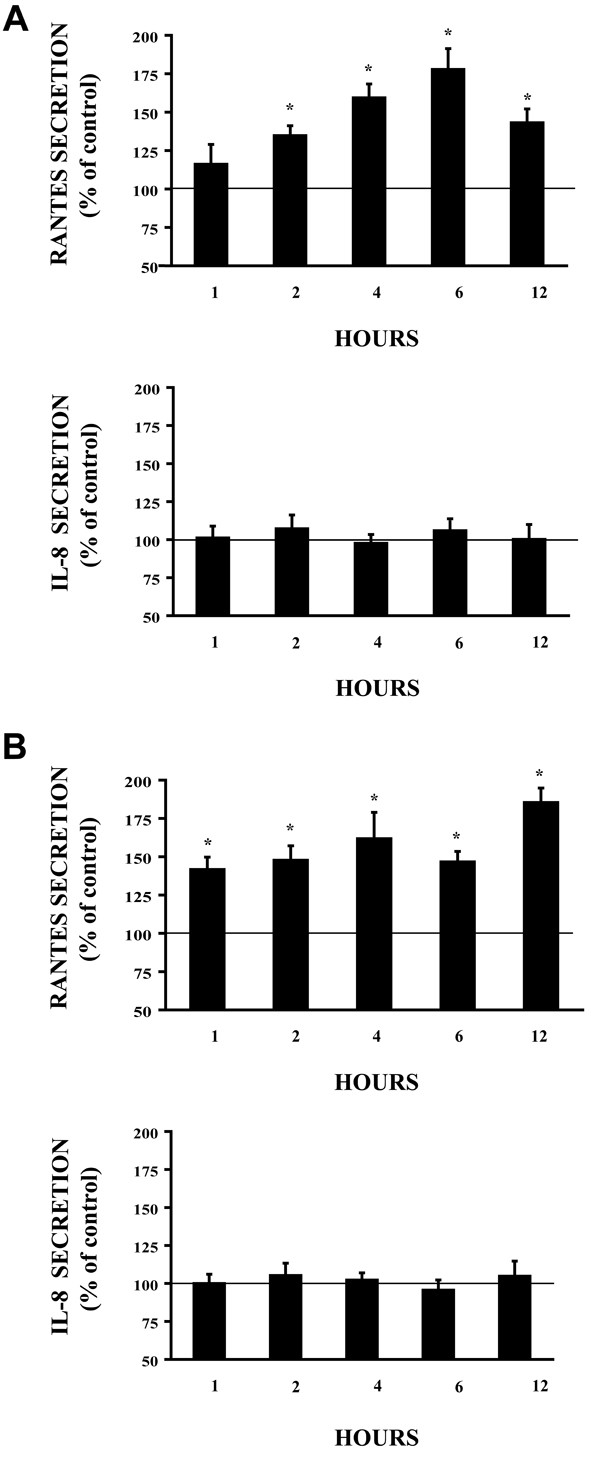
**Cross-linking of ICAM-1 induces selective chemokine secretion from airway epithelial cells. (Figure A. A549 cells) (Figure B. NHBE cells)**. Cultures were incubated with control IgG1 mAb and anti-mouse IgG F(ab')_2 _or anti-ICAM-1 mAb and anti-mouse IgG F(ab')_2 _for the indicated times. Both RANTES and IL-8 was detected in supernatants by ELISA.(n = 6; * = significantly different from control; p < 0.05).

To further investigate the effect of ICAM-1 cross-linking on both A549 cells and NHBE cells, we utilized an ELISA to determine the effect of ICAM-1 cross-linking on IL-8 and RANTES secretion. ICAM-1 cross-linking for 1, 2, 4, 6 and 12 hours demonstrated no effect on IL-8 secretion. However, in contrast to IL-8, cross-linking ICAM-1 stimulated RANTES secretion in both A549 cells (Fig. 3a) and NHBE cells (Fig 3b) in vitro. Once again, cultures that were not pretreated with TNFα to increase ICAM-1 surface expression failed to stimulate chemokine secretion when treated with antibodies to cross-link ICAM-1 and assayed by ELISA (data not shown).

### ICAM-1 induced RANTES production is mediated by p42/ p44 phosphorylation

**Figure 4:**
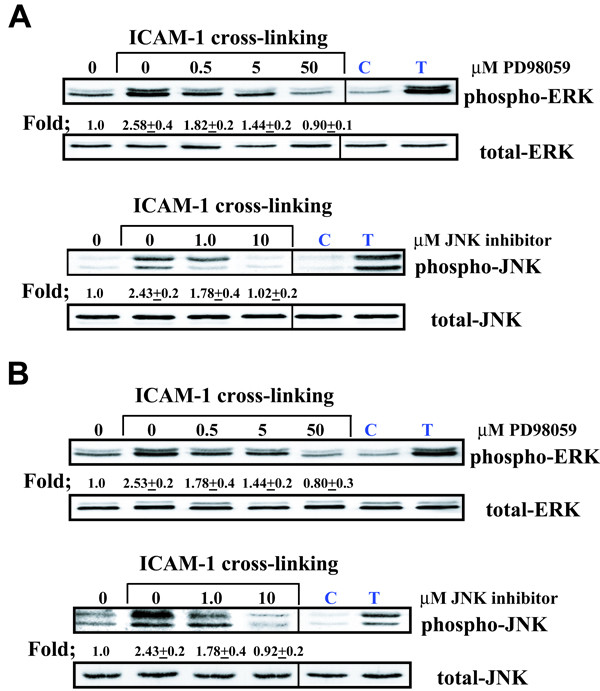
**Inhibition of ICAM-1 cross-linking induced phosphorylation of MAP kinases in airway epithelial cells (Figure A. A549 cells) (Figure B. NHBE cells)**. Cultures were pre-incubated in the presence of increasing concentrations of MAP kinase inhibitors. Cultures were then cross-linked for 10 minutes to assess ERK phosphorylation and 30 minutes to assess JNK phosphorylation. **Lane 0 **represents cultures that were cross-linked with control IgG1 mAb and anti-mouse IgG F(ab')_2 _for 10 minutes for ERK phosphorylation and 30 minutes for JNK phosphorylation. **Lane C **represents a negative protein control prepared from cells incubated in media alone. **Lane T **represents cultures that were stimulated with 10 mg/ml TNF for 10 min to serve as a positive control. Cultures that were not cross-liked and pre-incubated with either MAP kinase inhibitor alone had no effect on MAP Kinase phosphorylation. Fold increase in amounts of phosphorylated ERK and JNK proteins are expressed as the mean ± SD in three different experiments.

**Figure 5:**
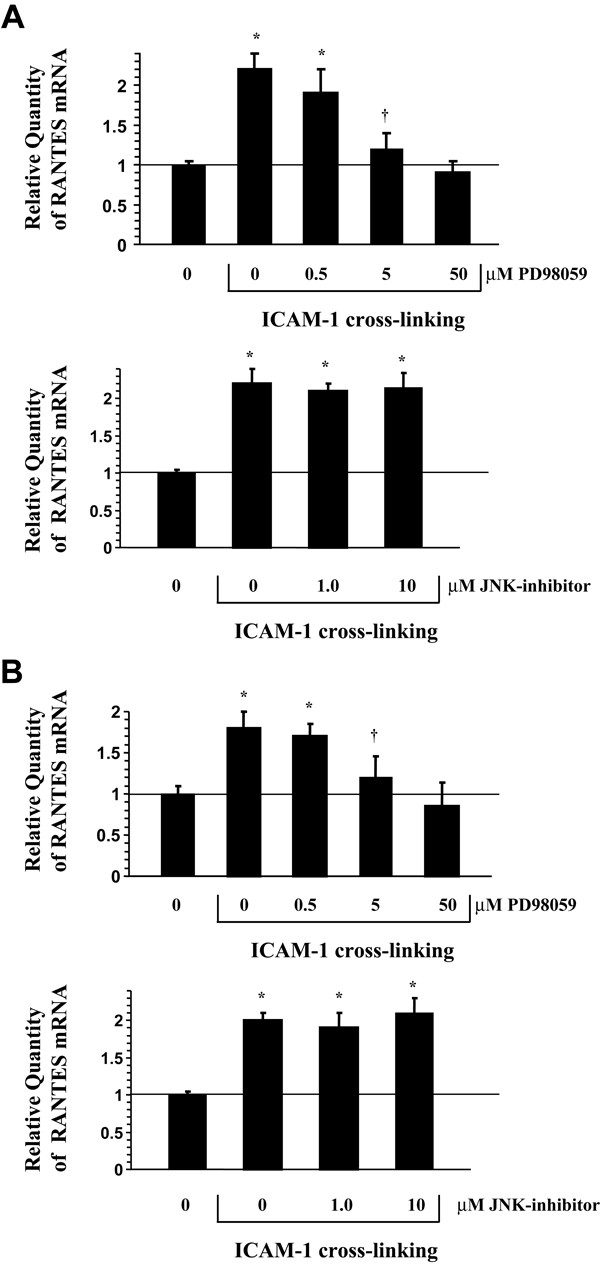
**Selective inhibition of ICAM-1 cross-linking induced RANTES mRNA in airway epithelial cultures by MAP kinase inhibitors (Figure A. A549 cells) (Figure B. NHBE cells)**. Cultures were pre-incubated in the presence of increasing concentrations of MAP kinase inhibitors and subsequently cross-linked for two hours with anti-ICAM-1 mAb and anti-mouse IgG F(ab')_2 _before RNA was isolated for RT-PCR analysis of RANTES message (40 PCR cycles). Messenger RNA of the 18s ribosomal gene was amplified under the same conditions and was used as the internal control. **Lane 0 **represents cultures that were cross-linked with control IgG1 mAb and anti-mouse IgG F(ab')_2 _for 2 hours. Cultures pre-incubated with MAP kinase inhibitors alone had no effect on RANTES or IL-8 mRNA. (n = 6; * = significantly different from control; † = significantly different from 0.5 μM PD98059; p < 0.05).

**Figure 6:**
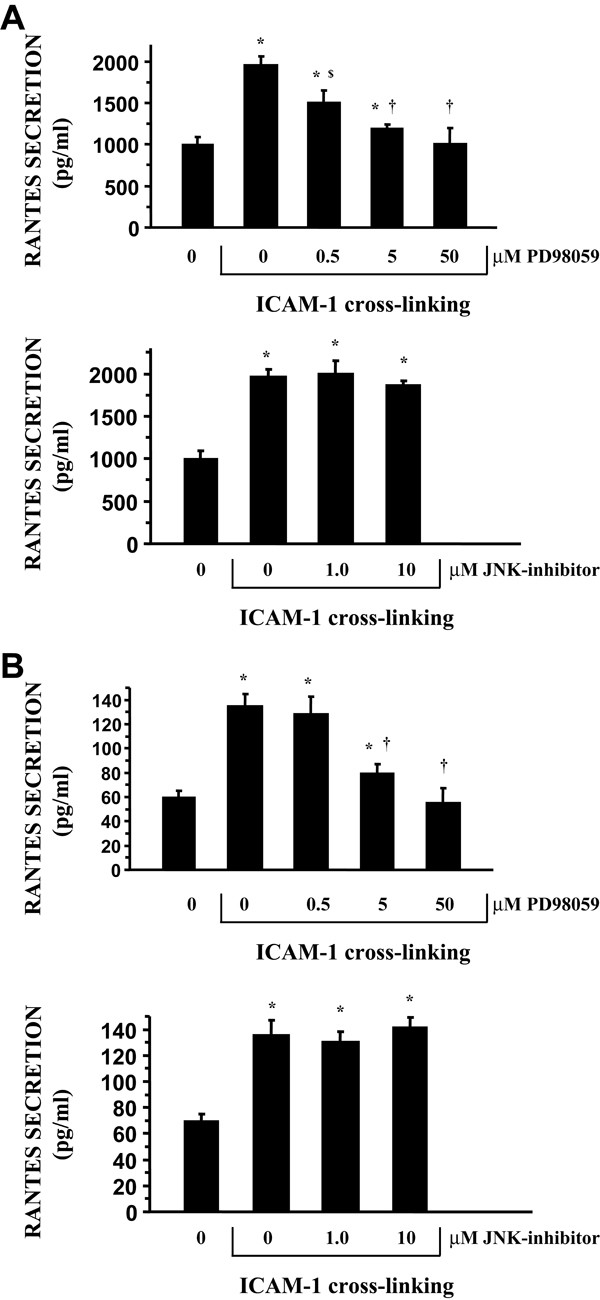
**Inhibition of ICAM-1 cross-linking induced RANTES secretion from airway epithelial cultures by PD98059 (Figure A. A549 cells) (Figure B. NHBE cells)**. Epithelial cultures were pre-incubated for 30 minutes in the presence of increasing concentrations of MAP kinase inhibitors. Cultures were then incubated with anti-ICAM-1 mAb and anti-mouse IgG F(ab')_2 _for 6 hours. Cultures pre-incubated with MAP kinase inhibitors alone had no effect on RANTES secretion. (Results represent means ± SD of triplicate measurements). (n = 6; * = significantly different from control; $ = significantly different from 0.0 μM PD98059; † = significantly different from 0.5 μM PD98059; p < 0.05).

Because ICAM-1 cross-linking stimulates phosphorylation of both p42 /p44 and JNK, we investigated the effect of the ERK inhibitor (PD98059) and a JNK inhibitor (JNK inhibitor-1) on RANTES regulation. As demonstrated by western blot analysis, PD98059 inhibited ICAM-1 induced p42/p44 phosphorylation in both A549 cells (Fig 4a.) and NHBE cells (Fig 4b.) in vitro. Additionally, JNK inhibitor-1 inhibited ICAM-1 induced JNK phosphorylation in both A549 cells and NHBE cells (Fig 4) in vitro. When examining the effect of these inhibitors on RANTES gene regulation, ICAM-1 cross-linking induced RANTES gene expression was inhibited by the ERK inhibitor PD98059 in A549 cells (Fig 5a.) and NHBE cells (Fig 5b.), while JNK inhibitor-1 had no effect on these events (Fig 5). In addition, PD98059 inhibited ICAM-1 induced RANTES secretion in both cultures and JNK inhibitor-1 had no effect on RANTES secretion in both cultures (Fig 6). Treatment of both cell cultures with PD98059 alone or JNK inhibitor-1 alone had no effect on chemokine gene expression or secretion at the doses and times indicated (data not shown).

### Cytotoxicity

None of the inhibitors or solubilization vehicles used in these studies affected RANTES gene expression, surface expression, or MAP kinase phosphorylation when added by themselves or in combination with treatments. In addition, all experiments were done with non-cytotoxic concentrations of TNFα and inhibitors as determined by LDH assay [26].

## Discussion

Intercellular adhesion molecule1 (ICAM-1) is an adhesion molecule that has multiple physiological functions including cell growth, differentiation, and leukocyte trafficking [2]. In the inflammatory process, ICAM-1 serves as the receptor for the Beta 2 integrin molecule present on leukocytes, promoting transendothelial migration to sites of inflammation [27]. In addition to diapedesis, ICAM-1 has been shown to function as a signaling molecule transmitting outside-inside signaling, resulting in multiple biological effects. Studies have demonstrated that activating ICAM-1 can induce phosphorylation of intracellular proteins, resulting in activation of both protein kinases and transcription factors [28-30].

The focus of this study was to determine whether ligation of ICAM-1 on airway epithelial cells previously exposed to the cytokine TNFα to increase ICAM-1 surface expression, resulted in outside-inside signaling. The technique of using antibodies to cross-link ICAM-1 has been utilized by numerous investigators attempting to discover the biological effects of ICAM-1 ligation; this ligation has been shown to mimic events that occur in vivo [22,31]. Cultures were pretreated with TNFα for two hours, then incubated in medium alone for 18 hours to up-regulate ICAM-1 surface expression. In these experiments we utilized two types of cell cultures; a human lung adenocarcinoma cell line (A549), as representative of distal respiratory epithelium, and primary cultures of normal human bronchial epithelial (NHBE) cells to serve as a representative of proximal respiratory epithelium.

Data presented here demonstrates for the first time that cross-linking ICAM-1 resulted in selective phosphorylation of MAP kinases in both A549 cells and a fully differentiated primary culture of NHBE cells. Surprisingly, cultures that were not exposed to TNFα, which express only basal levels of ICAM-1 on their surface [3] did not result in phosphorylation of MAP kinases, or an increase in cytokine gene expression and secretion. One possible explanation for these findings may be that basal or constitutive levels of ICAM-1 surface expression contain insufficient numbers of molecules on the cell surface preventing subsequent ICAM-1 dimerization preventing outside-inside signaling. Studies investigating HUVEC [32] and renal fibroblast [33]in vitro demonstrated that basal expression of ICAM-1 was sufficient to stimulate intracellular signaling. In contrast, cytokine stimulation was required on cardiomyocytes [22]to successfully alter cellular events related to cross-linking ICAM-1, suggesting ICAM-1 signaling events may appear to be dependent on the cell type or basal expression of ICAM-1. Currently, no studies have been reported that determine the minimum ICAM-1 molecules necessary for successful activation of ICAM-1 on the cell surface or how basal ICAM-1 surface expression differs between cell types. In these studies, cross-linking ICAM-1 in both airway cultures stimulated with TNFα to increase ICAM-1 surface expression, resulted in a selective increase in phosphorylation of ERK and JNK while having no effect on p38, as detected by western blot analysis utilizing antibodies directed specifically to phosphorylated p42/p44, JNK, and p38 in both cell cultures. Data presented here also demonstrated that phosphorylation of both ERK and JNK as a result of ICAM-1 cross-linking was inhibited by the commercially available MAP kinase inhibitors PD98059 and JNK inhibitor-1 respectively. PD98059 is a cell-permeable selective MAP kinase kinase (MEK) inhibitor that prevents activation of MAP kinase and subsequent phosphorylation of MAP kinase substrates. JNK activation was inhibited by a cell permeable biologically active peptide (JNK inhibitor-1), which inhibits the phosphorylation of the activation domains of JNK.

The upstream signaling mechanisms that induce phosphorylation and activation of MAP kinases following ICAM-1 ligation have yet to be determined and appear to be dependent on the cell type. The cytoplasmic domain of ICAM-1 is relatively short, contains two tyrosine residues, but does not contain the motif that has been shown to mediate Src family kinase, and has no apparent kinase activity [2]. Despite the lack of the Src motif, ligation of ICAM-1 on A20 B cell lymphoma cells has been shown to activate the Src family kinase p53/p56^*lyn*^, Raf-1 and MAP kinase ERK [28]. In addition, cross-linking ICAM-1 on human pulmonary microvascular endothelial cells induced activation of SRC tyrosine kinases through an oxidant dependent pathway that was dependent on xanthine oxidase [30]. How ICAM-1 ligation induces activation of these tyrosine kinases is currently unknown. However, in preliminary experiments, we have found that ICAM-1 cross-linking has been shown to stimulate tyrosine phosphorylation of several proteins in the airway epithelium (unpublished data).

Since ICAM-1 expression is upregulated in inflammation-associated lung diseases, and cross-linking ICAM-1 on airway epithelial activated ERK and JNK in vitro, we further investigated whether ICAM-1 cross-linking altered the expression of the pro-inflammatory chemokines RANTES and IL-8. As a member of the CC chemokine family of proteins, RANTES is a potent chemoattractant for eosinophils, basophils, monocytes, and memory T lymphocytes. As a member of the CXC chemokine family, IL-8 is also a potent chemoattractant for neutrophils. Both of these chemokines have been implicated in a variety of diseases characterized by lung inflammation [10,34,35]. Interestingly, cross-linking ICAM-1 in this study resulted in an increase in only RANTES gene expression and secretion and not IL-8 from both A549 cells and primary cultures of NHBE cells. Studies in human vascular endothelial cells (HUVEC) demonstrated that ICAM-1 activation resulted in enhanced RANTES gene expression and secretion [32], however this study also demonstrated an increase in IL-8 expression and secretion in HUVEC. The data presented in these studies investigating these chemokines demonstrate a different response in the airway epithelium. While cross-linking the airway epithelium resulted in an increase in RANTES gene expression and secretion, it did not increase either IL-8 gene expression or secretion. Additional studies investigating ICAM-1 cross-linking in human renal fibroblasts also stimulated RANTES expression, suggesting that the effects of cross-linking ICAM-1 appears to be cell specific [33]. For example, while both HUVEC [32] and renal fibroblasts [33] grown in culture are capable of stimulating RANTES expression following ICAM-1 ligation, HUVEC involved an ERK dependent pathway while renal fibroblasts utilized a calcium dependent, ERK independent signaling pathway. The upstream events leading to ERK phosphorylation following ICAM-1 cross-linking remains to be elucidated. In results presented here, both RANTES mRNA and protein secretion, induced by ICAM-1 cross-linking, were inhibited by PD98059 however unaltered by JNK inhibitor-1 in both airway epithelial cell cultures when analyzed with real-time PCR and ELISA respectively. Neither MAP kinase phosphorylation, RANTES mRNA, or secretion were induced in airway cultures when exposed to the following antibodies: an unrelated antibody such as VCAM-1, a control murine IgG1 antibody, a control murine IgG1 antibody and anti-mouse IgG(ab')_2_. These results demonstrated specificity to ICAM-1 ligation and not a response due to nonspecific alterations in the cells surface. Our data also indicates that ICAM-1 cross-linking stimulates a synergistic increase in TNFα-mediated RANTES production involving activation of ERK in airway epithelial cells.

In summary, we have demonstrated that cross-linking ICAM-1 on the surface of both A549 and NHBE cells selectively induced RANTES gene expression and protein secretion and did not alter either IL-8 gene expression or secretion. While A549 cells (lower airway) secreted substantially more RANTES than NHBE cells (upper airway), both cell types demonstrated that ICAM-1 cross-linking induced a synergistic increase in TNFα-mediated RANTES gene expression and protein secretion that is dependent on activation of MAP kinase p42/p44.

## Conclusion

There is increasing evidence demonstrating that intercellular adhesion molecule 1 is up-regulated in inflammatory diseases of the lung. Understanding the role of ICAM-1 in the lung is essential to understanding the pathogenesis of inflammatory airway diseases. The data from this study demonstrate for the first time that airway epithelial cells can selectively induce outside-inside signaling through ICAM-1 ligation. Specifically, cross-linking ICAM-1 stimulated RANTES gene expression and protein secretion through an ERK-dependent pathway. These results suggest that ICAM-1 can function not only as a receptor for diapedesis of leukocytes, but may also enhance inflammation in the airway through the secretion of chemokines such as RANTES.

## Abbreviations

RANTES, regulated on activation normal T cell expressed and secreted; NHBE, normal human bronchial epithelial; TNFα, tumor necrosis factor alpha; ICAM-1, intercellular adhesion molecule 1; BEGM, bronchial epithelial cell growth medium; DMEM-H, Dulbecco's modified Eagle's medium with high glucose; EGF, epidermal growth factor; MAP, mitogen-activated protein; MEK, MAP kinase kinase; PBS, phosphate buffered saline; RT-PCR, reverse transcriptase-polymerase chain reaction; mAb, monoclonal antibody.

## Authors' contributions

TMK conceived the study, formulated its design and coordination, performed the experiments and drafted the manuscript. CLJ initiated and maintained cell cultures, collected samples during experimental procedures, performed western blots and ELISAs, and participated in preparing the manuscript. All authors read and approved the final manuscript.
